# Metabolic Unhealthiness is Associated With Increased Risk of Critical COVID-19 Pneumonia and Inpatient Mortality in Hospitalized Patients with Obesity or Overweight

**DOI:** 10.7759/cureus.42205

**Published:** 2023-07-20

**Authors:** Pedro Cortés, Paul Travers, Jennifer J Zeng, Colleen T Ball, Scott A Lynch, Victoria Gómez

**Affiliations:** 1 Internal Medicine, Mayo Clinic, Jacksonville, USA; 2 Neuroscience, Krieger School of Arts and Sciences, Johns Hopkins University, Baltimore, USA; 3 Clinical Trials and Biostatistics, Mayo Clinic, Jacksonville, USA; 4 Bariatric Surgery, Mayo Clinic, Jacksonville, USA; 5 Gastroenterology, Mayo Clinic, Jacksonville, USA

**Keywords:** morbidity and mortality, risk calculators, metabolic disease, comorbid obesity, covid-19 pneumonia

## Abstract

Background and aims

Being metabolically unhealthy (MU) is defined as having either hypertension, hyperlipidemia, type 2 diabetes mellitus/pre-diabetes, or fatty liver disease. We aimed to determine if MU was associated with severe COVID-19 pneumonia (severe disease).

Methods

We performed a single-center retrospective study between March 2020 and August 2021 for patients with overweight or obesity hospitalized with COVID-19 pneumonia. Logistic regression analysis was utilized to derive a risk score for severe disease. The accuracy of the model was assessed using the area under the receiver operating characteristic curve (AUROCC) and bootstrap resampling.

Results

A total of 334 of 450 patients hospitalized with COVID-19 pneumonia (74.2%) were MU. Patients who were MU had higher in-hospital mortality (10.5% vs. 2.6%) and longer length of hospitalization (median 6 vs. 4 days). MU was not associated with severe disease, p=0.311. On multivariable analysis, older age, male sex, and Asian race were associated with severe disease. Not being vaccinated was associated with doubled odds of severe disease. The AUROCC of the final model was 0.66 (95% CI: 0.60 to 0.71). The risk score at the lowest quintile had a 33.1% to 65.5% predicted risk and a 58.7% observed risk of severe disease, whereas, at the highest quintile, there was an 85.7% to 97.7% predicted risk and an 89.7% observed risk of severe disease.

Conclusion

Being MU was not a predictor of severe disease, even though mortality was higher despite having higher rates of vaccination. This risk score may help to predict severe disease in hospitalized patients with obesity or overweight. External validation is recommended.

## Introduction

COVID-19 pneumonia, caused by the SARS-CoV-2 virus, emerged in late 2019 and quickly became a global pandemic despite quarantine efforts [1]. Multiple studies have shown that obesity, and certain metabolic conditions, such as type 2 diabetes mellitus (T2DM), are associated with severe COVID-19 pneumonia [2-6]. Additionally, several risk scores have been developed to predict in-hospital mortality, severe disease progression, and adverse outcomes from COVID-19 [7-9].

The concept of metabolic health has started to emerge within the past few years [10,11]. Metabolic conditions include pre-diabetes, T2DM, hypertension, hyperlipidemia, and fatty liver. Although the definitions of metabolic unhealthiness vary, it is generally defined as having at least one metabolic condition [10, 12]. It is posited that metabolic unhealthiness increases the risk of disease severity in multiple conditions as not all fat mass carries the same risk of complications [12]. A recent study, for example, showed that being metabolically unhealthy increased the likelihood of progression to liver fibrosis in patients with fatty liver [13]. Regarding COVID-19 pneumonia, few studies have examined the role of metabolic health and disease severity [14].

Although obesity is an established risk factor for severe COVID-19 pneumonia, it remains unclear whether metabolic health plays a role as a risk factor. Therefore, we aimed to determine the association between being metabolically unhealthy with severe or critical COVID-19 pneumonia, and in-hospital mortality in patients with overweight or obesity. Finally, we aimed to derive risk scores to predict severe disease and critical illness from COVID-19 pneumonia in this patient population. We hypothesized metabolic unhealthiness would be predictive of severe, critical, and inpatient mortality from COVID-19 pneumonia.

## Materials and methods

Patient selection

The current study was approved by the Mayo Clinic Institutional Review Board. A waiver of informed consent was obtained. The study followed the STROBE guidelines for observational studies, which has been provided as a supplementary material [15].

Patients hospitalized with COVID-19 pneumonia between March 2020 and August 2021 were identified using our institution’s registry of confirmed cases. A random selection of 450 adult patients, aged >/= 18 years of age, from the registry were enrolled. Patients were excluded from the cohort for the following reasons: 1) having a body mass index (BMI) less than 25.0 kg/m^2^, and 2) being immunocompromised (defined as being a solid organ or bone marrow transplant recipient, receiving immunosuppressive medications or chemotherapy, or having a history of an immunocompromised condition, such as human immunodeficiency virus (HIV), severe combined immunodeficiency, etc. Patients with a normal BMI were excluded to determine the impact of metabolic health, specifically, in patients with excess fat mass. A BMI >/= 30.0 kg/m^2 ^was defined as having obesity. Class I, II, and III obesity were defined as a BMI between 30.0 to 34.99 kg/m^2^, 35.00 to 39.99 kg/m^2^, and >/= 40.00 kg/m^2^, respectively. Likewise, patients who were immunocompromised were excluded as they represent a different subset of patients who likely have worse outcomes. All patients had follow-up data from admission to discharge from the hospital.

Primary and secondary outcomes

Severe COVID-19 pneumonia (onward referred to as severe disease) was defined as per the National Institutes of Health (NIH) COVID-19 treatment guidelines, including 1) oxygen saturation < 94 % on room air, 2) a P:F (PaO2 (partial pressure of oxygen in the arterial blood)/FiO2 (fraction of inspired oxygen)) ratio < 300, 3) respiratory rate > 30, and 4) the presence of greater than 50% of lung infiltrates on chest imaging [16]. The primary outcome was the development of severe disease during the hospitalization for COVID-19 pneumonia.

Secondary outcomes included in-hospital mortality from COVID-19 pneumonia, and other measures of disease severity, including admission to the intensive care unit (ICU), need for endotracheal intubation, initiation of vasopressors, development of atrial fibrillation with rapid ventricular response, initiation of continuous renal replacement therapy (CRRT), need for extracorporeal membrane oxygenation (ECMO), and length of hospital stay. The NIH guidelines define critical illness as respiratory failure, septic shock, and/or multiple organ dysfunction [16]. A secondary composite outcome was employed and included all binary secondary outcomes except death. This secondary composite outcome was termed critical illness. 

Risk score development and statistical analysis

All variables were collected retrospectively. Demographic data included age at admission, sex category (male or female), race and ethnicity, smoking status, and BMI. The presence of metabolic disorders prior to admission was recorded and included hypertension, hyperlipidemia, T2DM or pre-diabetes, and the presence of fatty liver. The presence of hypertension, hyperlipidemia, and T2DM was determined through ICD-10-CM codes and a retrospective review of clinical notes. A diagnosis of pre-diabetes was determined by a hemoglobin A1C between 5.8 to 6.4%. The presence of fatty liver was determined by a review of prior abdominal imaging reports. Having at least one metabolic disorder was defined as being metabolically unhealthy (MU).

The presence of pulmonary disease was recorded and included obstructive sleep apnea, asthma, chronic obstructive pulmonary disease (COPD), or other conditions. The other conditions included prior histories of lobectomy, interstitial lung disease or idiopathic pulmonary fibrosis, or pulmonary hypertension. Treatments intended to target COVID-19 were recorded and included remdesivir, convalescent plasma, immunomodulators (tocilizumab, baricitinib, and lenzilumab), and corticosteroids (methylprednisolone, dexamethasone, etc.).

The selection criteria for the varied treatment options changed over time during the study period as new therapies emerged during the COVID-19 pandemic. In general, however, remdesivir was given to all patients without a contraindication (such as an estimated glomerular filtration rate (eGFR) < 30 mL/min or elevated transaminases), corticosteroids for patients with an oxygen requirement of > 4 L per nasal cannula, the use of convalescent plasma in patients with non-reactive antibody titers to the spike antigen, and the use of immunomodulators for refractory cases with evidence of cytokine release storm.

Baseline characteristics were reported using descriptive statistics summarized as medians and interquartile ranges, or fractions and percentages for continuous and categorical variables, respectively. Wilcoxon Rank Sum and Fisher’s Exact tests were utilized to determine associations with metabolic status. Multivariable logistic regression was performed to adjust for potential confounders and to identify predictors for severe disease. Variables with the highest p-values were sequentially removed until removal led to less than a 1-point reduction in the Akaike information criterion (AIC). The accuracy of the model was calculated using bootstrap resampling estimates of the area under the receiver operating characteristic curve (AUROCC). Secondary analyses for critical illness and in-hospital mortality were performed utilizing bivariable logistic regression analyses. A separate risk score for critical illness was also derived through the same method. 

All tests performed were two-sided, and the statistical significance was set at a p-value < 0.05. Using an alpha of 0.05, a beta of 0.80, and a baseline incidence of critical COVID-19 pneumonia of 20% in patients with obesity, we estimated a sample size of 398 patients would be needed to show a 10% reduction in the rate of critical illness in metabolically healthy patients. SPSS Statistics for Windows, Version 28.0 (Armonk, USA: IBM Corp) was used for the statistical analyses.

## Results

Patient characteristics

**Figure 1:**
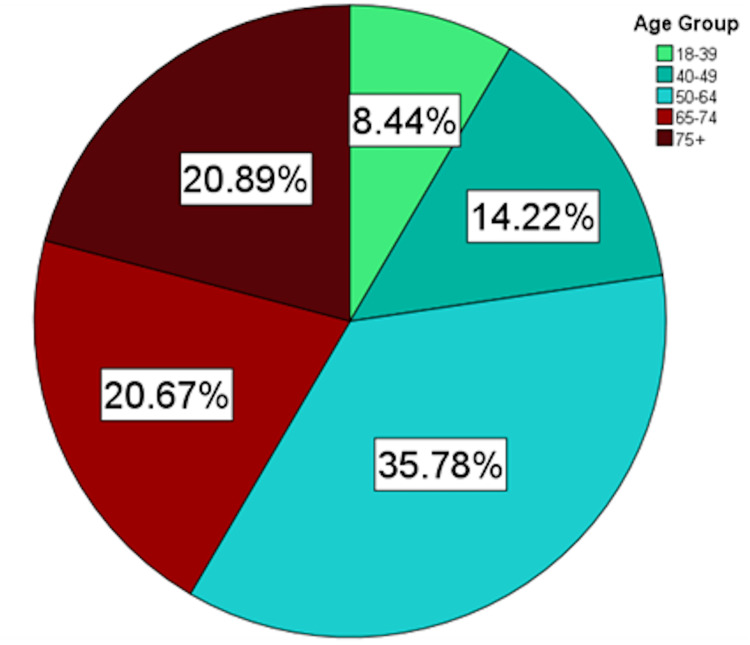
Age Distribution of Patients Hospitalized with COVID-19 Pneumonia COVID-19, coronavirus disease of 2019

**Figure 2:**
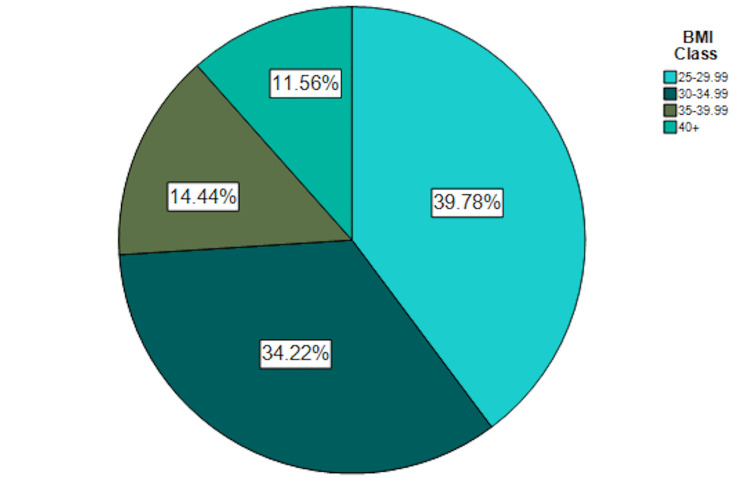
Body Mass Index Classification of Patients Hospitalized with COVID-19 Pneumonia COVID-19, coronavirus disease of 2019

**Figure 3:**
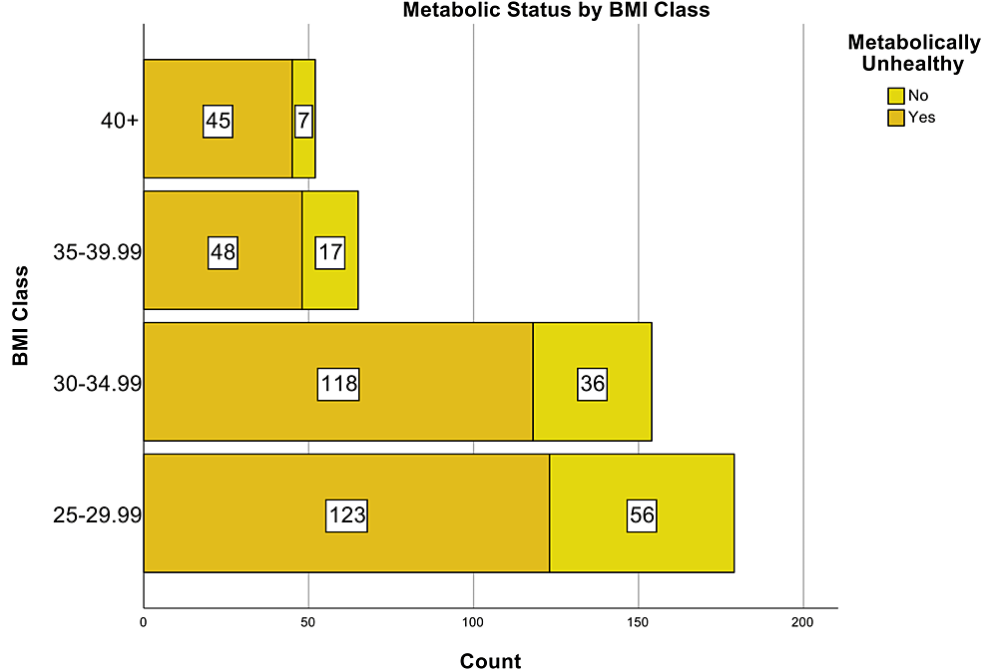
Metabolic Status by BMI Class in Patients Hospitalized with COVID-19 Pneumonia BMI, body mass index; COVID-19, coronavirus disease of 2019

A total of 450 patients with overweight or obesity hospitalized with COVID-19 pneumonia were included in the derivation of the risk score (Table 1). The median age at hospitalization was 61.7 years (interquartile range [IQR]: 51.2 - 72.7 years) and 62.4% were male. About half of the patients were between the ages of 40 - 64 years (Figure 1). The median BMI was 31.1 kg/m2 (28.0 - 35.1 kg/m2) and about 75% of patients were between 25.0 to 34.99 kg/m2 (Figure 2). 74.2% of patients were considered MU. The most common metabolic conditions were hypertension (64.4%) and hyperlipidemia (50.9%). The fraction of patients considered MU increased with increasing BMI (Figure 3). A total of 343 out of 450 patients (76.2%) had severe disease. The in-hospital mortality rate from COVID-19 pneumonia was 8.4%.

**Table 1:** Observed Proportion of Critical Illness from COVID-19 Pneumonia According to the Sum of the Score Coefficients COVID-19, coronavirus disease of 2019; 95% CI, 95% confidence interval

Sum of Score Coefficient	Proportion Critical COVID-19 Pneumonia (95% CI)
[-2.4193, -2.0645)	0.082 (0.027, 0.181)
[-2.0645]	0.113 (0.063, 0.182)
(-2.0645, -1.6631]	0.149 (0.082, 0.242)
(-1.6631, -1.290]	0.207 (0.138, 0.289)
(-1.290, 0.389]	0.281 (0.170, 0.415)

Derivation of risk score

Age, male sex, hypertension, T2DM, and BMI group 40.0 kg/m2 or greater were the strongest predictors of severe disease on bivariable logistic regression (Table 2). Being MU was not associated with severe disease, p=0.311. However, every 1 increase in the number of metabolic conditions increased the likelihood of severe disease by 24%, (OR 1.24, p = 0.0341). After inputting these variables into a multivariable model, and subsequently removing those with the highest p-values, a nine-variable model was constructed (Table 3). In this model, the three strongest predictors for severe disease included age between 65-74 years (OR 2.59, p = 0.0396), male sex (OR 1.64, p = 0.0324), and Asian race (OR 4.49, p = 0.0482). Not being vaccinated was associated with a 124% increased likelihood of severe disease (OR 2.24, p = 0.0282). 

**Table 2:** Baseline Characteristics of All Patients Hospitalized with COVID-19 Pneumonia by Metabolic Status IQR, interquartile range; COPD, chronic obstructive pulmonary disease; ICU, intensive care unit; CRRT, continuous renal replacement therapy; ECMO, extracorporeal membrane oxygenation 1. Wilcoxon Rank Sum Test; 2. Fisher’s Exact Test

Median (IQR) or Fraction (%)	All Patients N=450	Metabolically Healthy N=116	Metabolically Unhealthy N=334	P value
Age, years	61.7 (51.2-72.7)	54.4 (46.2-64.4)	64.1 (53.8-74.0)	<0.001^1^
· Age Group 18-39	38 (8.4%)	22 (19.0%)	16 (4.8%)	
· Age Group 40-49	64 (14.2%)	22 (19.0%)	42 (12.6%)	
· Age Group 50-64	161 (35.8%)	43 (37.1%)	118 (35.3%)	
· Age Group 65-74	93 (20.7%)	19 (16.4%)	72 (22.2%)	
· Age Group 75+	94 (20.9%)	10 (8.6%)	84 (25.1%)	
Male sex	281 (62.4%)	72 (62.1%)	209 (62.6%)	1.000^2^
Race				0.589^2^
· White Race	355/444 (80.0%)	87 (76.3%)	268 (81.2%)	
· Black Race	55/444 (12.4%)	16 (14.0%)	39 (11.8%)	
· Asian Race	26/444 (5.9%)	9 (7.9%)	17 (5.2%)	
· Other	8/444 (1.8%)	2 (1.8%)	6 (1.8%)	
Hispanic Ethnicity	25 (5.6%)	5 (4.3%)	20 (6.0%)	0.640^2^
Smoking Status				0.006^2^
· Never Smoker	283 (62.9%)	87 (75.0%)	196 (58.7%)	
· Current Smoker	16 (3.6%)	3 (2.6%)	13 (3.9%)	
· Former Smoker	151 (33.6%)	26 (22.4%)	125 (37.4%)	
Body Mass Index, kg/m^2^	31.1 (28.0-35.1)	30.1 (27.8-34.1)	31.5 (28.2-35.6)	0.027^1^
Body Mass Index Classification				0.056^2^
· 25.00-29.99	179 (39.8%)	56 (48.3%)	123 (36.8%)	
· 30.00-34.99	154 (34.2%)	36 (31.0%)	118 (35.3%)	
· 35.00-39.99	65 (14.4%)	17 (14.7%)	48 (14.4%)	
· 40.00+	52 (11.6%)	7 (6.0%)	45 (13.5%)	
Vaccinated for SARS-CoV-2	42 (9.3%)	4 (3.4%)	38 (11.4%)	0.009^2^
Metabolic Disorders				
Hypertension	290 (64.4%)	NA	NA	NA
Hyperlipidemia	229 (50.9%)	NA	NA	NA
Type 2 Diabetes Mellitus	119 (26.4%)	NA	NA	NA
Pre-Diabetes	16 (3.6%)	NA	NA	NA
Fatty Liver	14 (3.1%)	NA	NA	NA
Metabolically Unhealthy	334 (74.2%)	NA	NA	NA
Number of Metabolic Disorders		NA	NA	NA
1	109 (32.6%)	NA	NA	NA
2	125 (37.4%)	NA	NA	NA
3	91 (27.2%)	NA	NA	NA
4	9 (2.7%)	NA	NA	NA
Pulmonary Disease				
Pulmonary Disease Present	128 (28.4%)	30 (25.9%)	98 (29.3%)	0.551^2^
· Obstructive Sleep Apnea	72 (56.3%)	15 (50.0%)	57 (58.2%)	0.529^2^
· Asthma	36 (28.1%)	15 (50.0%)	21 (21.4%)	0.005^2^
· COPD	24 (18.8%)	4 (13.3%)	20 (20.4%)	0.593^2^
· Other	12 (9.4%)	1 (3.3%)	11 (11.2%)	0.292^2^
Treatments Received				
Remdesivir	417 (92.7%)	107 (92.2%)	310 (92.8%)	0.837^2^
Convalescent Plasma	276 (61.3%)	72 (62.1%)	204 (61.1%)	0.912^2^
Corticosteroids	304 (67.6%)	75 (64.7%)	229 (68.6%)	0.490^2^
Immunomodulators	95 (21.1%)	26 (22.4%)	69 (20.7%)	0.693
Outcomes				
Severe COVID-19 Pneumonia	343 (76.2%)	84 (72.4%)	259 (77.5%)	0.311^2^
In-hospital Mortality	38 (8.4%)	3 (2.6%)	35 (10.5%)	0.006^2^
Secondary Outcome, Composite	73 (16.2%)	15 (12.9%)	58 (17.4%)	0.308^2^
· Admitted to ICU	67 (14.9%)	14 (12.1%)	53 (15.9%)	0.366^2^
· Intubated	34 (7.6%)	6 (5.2%)	28 (8.4%)	0.312^2^
· Vasopressors Required	39 (8.7%)	6 (5.2%)	33 (9.9%)	0.130^2^
· Atrial Fibrillation with RVR	20 (4.4%)	3 (2.6%)	17 (5.1%)	0.309^2^
· CRRT	10 (2.2%)	2 (1.7%)	8 (2.4%)	1.000^2^
· ECMO	5 (1.1%)	1 (0.9%)	4 (1.2%)	1.000^2^
Length of stay, days	5 (4-9)	4 (3-5)	6 (4-11)	<0.001^1^

**Table 3:** Bivariable Logistic Regression to Predict Severe or Critical COVID-19 Pneumonia, and In-hospital Mortality in Hospitalized Patients with Overweight or Obesity OR, odds ratio; CI, confidence interval; BMI, body mass index; COPD, chronic obstructive pulmonary disease; ICU, intensive care unit; NA, not applicable (for reference variables and for estimates of odds ratio that were not possible, including for four metabolic conditions under severe disease, and for asthma under in-hospital mortality due to zero cell counts)

Independent Variables	Bivariable Logistic Regression					
	Severe Disease		Critical Illness		In-hospital Mortality	
	OR (95% CI)	P value	OR (95% CI)	P value	OR (95% CI)	P value
Age, per 10 years	1.17 (1.00-1.35)	0.0401	1.31 (1.10-1.56)	0.0027	1.60 (1.26-2.05)	0.0002
Age Group 18-39 (reference)	1.00 (ref.)	NA	1.00 (ref.)	NA	1.00 (ref.)	NA
Age Group 40-49	1.06 (0.46-2.49)	0.88	1.21 (0.28-5.13)	0.80	1.19 (0.10-13.62)	0.89
Age Group 50-64	1.74 (0.81-3.74)	0.15	2.25 (0.64-7.85)	0.21	2.45 (0.30-19.75)	0.40
Age Group 65-74	2.50 (1.06-5.91)	0.0365	2.24 (0.61-8.25)	0.22	4.46 (0.55-36.11)	0.16
Age Group 75+	1.92 (0.84-4.42)	0.1234	3.78 (1.06-13.45)	0.0401	7.03 (0.89-55.21)	0.0638
Male	1.57 (1.01-2.44)	0.0447	1.48 (0.86-2.55)	0.15	1.53 (0.74-3.16)	0.26
Race, White (reference)	1.00 (ref.)	NA	1.00 (ref.)	NA	1.00 (ref.)	NA
Race, Black	0.64 (0.35-1.18)	0.15	0.61 (0.25-1.50)	0.29	0.65 (0.19-2.21)	0.49
Race, Asian	3.72 (0.86-16.07)	0.0785	1.19 (0.43-3.29)	0.73	2.04 (0.66-6.33)	0.22
Race, Other	2.17 (0.26-17.89)	0.47	1.67 (0.33-8.49)	0.54	3.75 (0.72-19.41)	0.12
Hispanic	1.26 (0.26-3.45)	0.65	1.31 (0.48-3.62)	0.60	0.44 (0.06-3.32)	0.42
Body Mass Index, per 5 kg/m^2^	1.10 (0.92-1.30)	0.31	0.86 (0.70-1.07)	0.18	0.67 (0.47-0.96)	0.0279
BMI Group 25-29.99 (reference)	1.00 (ref.)	NA	1.00 (ref.)	NA	1.00 (ref.)	NA
BMI Group 30-34.99	1.10 (0.67-1.82)	0.71	0.75 (0.42-1.34)	0.33	0.55 (0.26-1.17)	0.12
BMI Group 35-39.99	0.76 (0.40-1.41)	0.38	0.60 (0.26-1.37)	0.22	0.23 (0.05-0.99)	0.0488
BMI Group 40.00+	2.57 (1.03-6.43)	0.0429	0.78 (0.33-1.80)	0.55	0.44 (0.13-1.52)	0.19
Never Smoker (reference)	1.00 (ref.)	NA	1.00 (ref.)	NA	1.00 (ref.)	NA
Current Smoker	1.04 (0.33-3.34)	0.94	2.34 (0.78-7.06)	0.13	1.69 (0.36-7.94)	0.50
Former Smoker	1.40 (0.87-3.34)	0.17	0.88 (0.51-1.53)	0.65	1.21 (0.60-2.44)	0.59
Pulmonary Disease	1.61 (0.96-2.70)	0.0696	0.94 (0.54-1.65)	0.83	1.03 (0.49-2.14)	0.94
Obstructive Sleep Apnea	1.87 (0.75-4.66)	0.18	0.58 (0.22-1.53)	0.27	0.62 (0.18-2.15)	0.45
Asthma	0.68 (0.26-1.78)	0.44	0.11 (0.01-0.85)	0.0347	NA	NA
COPD	1.12 (0.34-3.65)	0.85	2.88 (1.00-8.28)	0.0492	2.77 (0.74-10.37)	0.13
Vaccinated	0.52 (0.27-1.03)	0.0597	1.71 (0.80-3.66)	0.17	2.43 (1.00-5.92)	0.0504
Metabolically Unhealthy	1.32 (0.81-2.13)	0.26	1.41 (0.77-2.61)	0.27	4.41 (1.33-14.62)	0.0153
Number of metabolic conditions						
0 (reference)	1.00 (ref.)	NA	1.00 (ref.)	NA	1.00 (ref.)	NA
1	1.10 (0.61-2.00)	0.75	1.07 (0.50-2.32)	0.85	3.39 (0.98-15.59)	0.0729
2	1.11 (0.62-1.97)	0.73	1.28 (0.62-2.64)	0.50	4.37 (1.37-19.44)	0.0241
3	1.93 (0.99-3.92)	0.0608	1.78 (0.85-3.73)	0.13	4.13 (1.19-19.06)	0.0375
4	NA	NA	5.39 (1.30-22.34)	0.0203	30.1 (5.4-194.6)	<0.001
Metabolic Conditions, per 1 condition	1.24 (1.02-1.50)	0.0341	1.28 (1.03-1.61)	0.0281	1.63 (1.21-2.25)	0.0017
Hypertension	1.59 (1.02-2.48)	0.0391	1.34 (0.78-2.30)	0.29	2.19 (0.98-4.90)	0.0560
Hyperlipidemia	1.13 (0.73-1.74)	0.59	1.47 (0.89-2.45)	0.14	2.23 (1.10-4.54)	0.0269
Pre-Diabetes	0.93 (0.30-2.96)	0.91	0.73 (0.16-3.28)	0.68	0.72 (0.09-5.57)	0.75
Type 2 Diabetes	1.76 (1.03-3.03)	0.0389	1.69 (0.99-2.88)	0.0539	1.93 (0.97-3.84)	0.0605
Fatty Liver	4.18 (0.54-32.3)	0.17	4.13 (1.39-12.29)	0.0108	9.47 (3.10-28.96)	<0.0001
Remdesivir	5.07 (2.45-10.52)	<0.0001	0.58 (0.25-1.34)	0.20	0.64 (0.21-1.94)	0.43
Convalescent Plasma	2.70 (1.73-4.21)	<0.0001	2.57 (1.42-4.64)	0.0017	2.53 (1.13-5.66)	0.0237
Corticosteroids	17.86 (10.36-30.78)	<0.0001	8.13 (3.20-20.63)	<0.0001	6.20 (1.87-20.51)	<0.0001
Immunomodulator	4.21 (1.97-9.00)	0.0002	2.88 (1.68-4.94)	0.0001	3.07 (1.54-6.11)	0.0014

Accuracy of the risk score for severe disease

**Figure 4:**
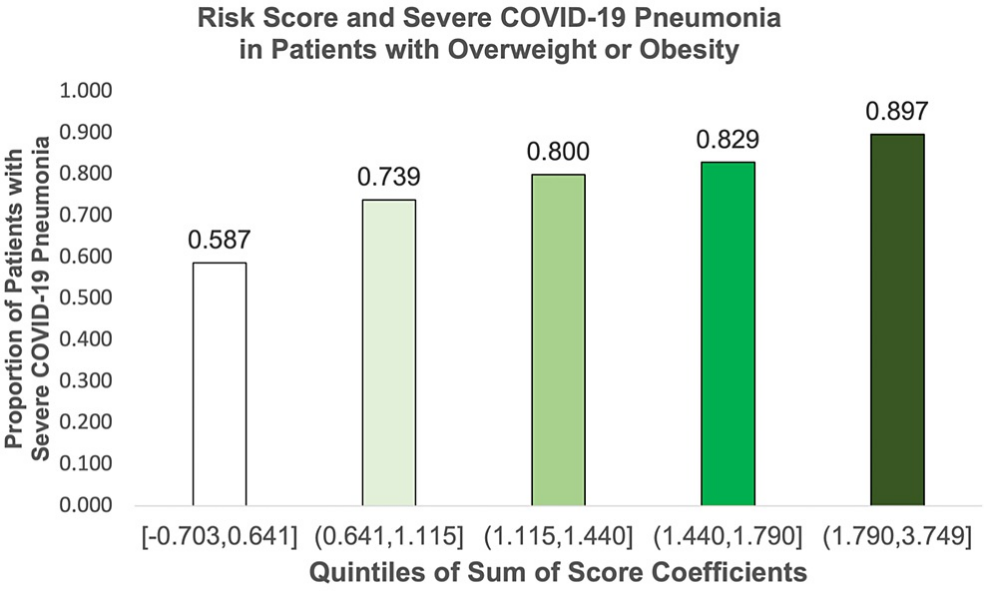
Accuracy of the Risk Score to Predict Severe COVID-19 Pneumonia in Hospitalized Patients with Overweight or Obesity COVID-19, coronavirus disease of 2019

The sum of the score coefficients was subdivided into quintiles and the observed risk for severe disease was calculated for our cohort (Table 4). The predicted risk of severe disease at the lowest quintile was 33.1% to 65.5%, and the observed risk was 58.7% (95% CI: 47.9 - 69.8%). At the highest quintile, the predicted risk was 85.7% to 97.7%, and the observed risk was 89.7% (95% CI: 79.9 - 95.9%). The AUROCC of the model was 0.66 (95% CI: 0.60 to 0.71) (Figure 4).

**Table 4:** Multivariable Logistic Regression to Derive a Risk Score to Predict Severe COVID-19 Pneumonia in Hospitalized Patients with Overweight or Obesity Calculated Predicted Risk =   2.718 ^ (sum of score coefficient) / (1 + 2.718 ^ (sum of score coefficients)), AIC: 479.18, AUROCC: 0.658 (95%: 0.60 – 0.71) AIC: Akaike information criterion; AUROCC: area under the receiver operating characteristic curve

	OR (95% CI)	P Value	Score Coefficient
(Intercept)	1.13 (0.53-2.51)	0.749	+ 0.127
Age Group 40-49	0.98 (0.40-2.34)	0.958	- 0.0235
Age Group 50-65	1.64 (0.73-3.57)	0.219	+ 0.494
Age Group 65-74	2.59 (1.04-6.42)	0.0396	+ 0.950
Age Group 75	1.98 (0.82-4.74)	0.126	+ 0.683
Male	1.64 (1.04-2.59)	0.0324	+ 0.495
Race, Asian	4.49 (1.25-28.8)	0.0482	+ 1.50
Vaccinated	0.45 (0.22-0.93)	0.0282	- 0.808
Hypertension	1.39 (0.86-2.26)	0.180	+ 0.332
Type 2 Diabetes	1.40 (0.80-2.55)	0.248	+ 0.340

In-hospital mortality and critical illness

Older age, being a former cigarette smoker, having a higher BMI, having been vaccinated, and having lower rates of asthma were associated with being MU. Patients who were MU had a higher rate of in-hospital mortality from COVID-19 pneumonia (10.5% vs. 2.6%, p = 0.006), and longer length of hospitalization (median 6, IQR: 4-11 vs. 4 IQR: 3-5, p < 0.001) than those who were metabolically healthy (MH) (Table 1). The secondary composite outcome, i.e., critical illness, was not associated with being MU, p = 0.308.

**Figure 5:**
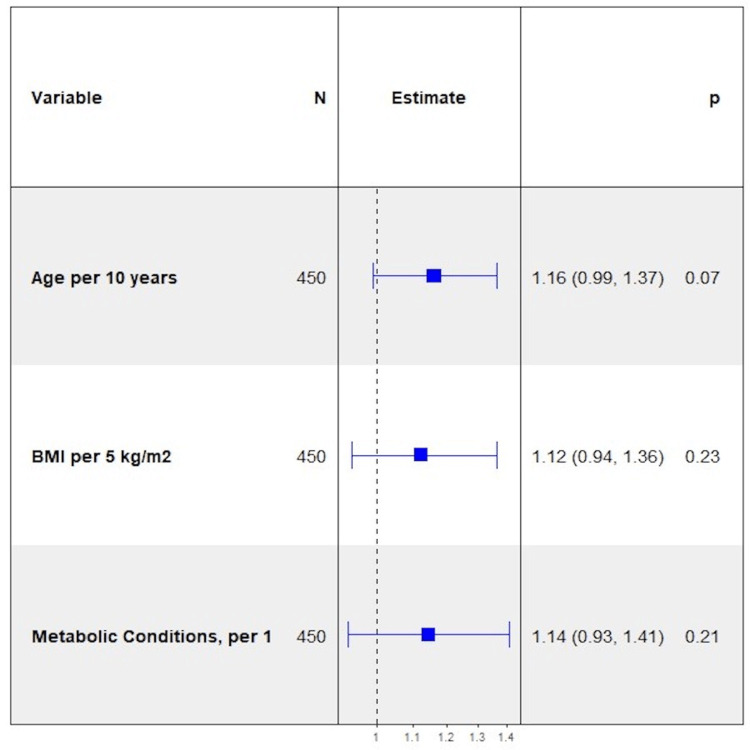
Forest Plot of Multivariable Logistic Regression Model for Severe Disease - Adjusted for Age and Body Mass Index.

**Figure 6:**
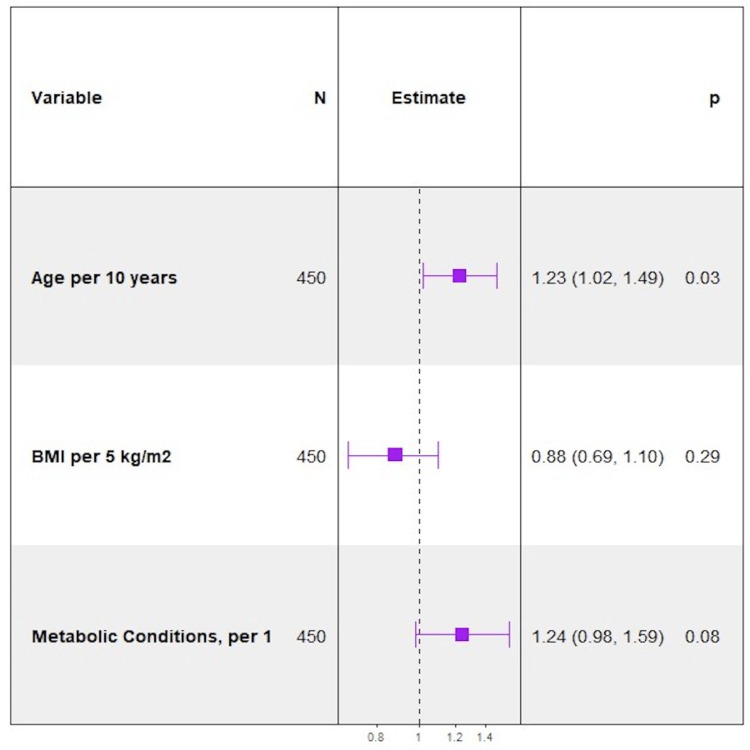
Forest Plot of Multivariable Logistic Regression Model for Severe Disease - Adjusted for Age and Body Mass Index.

**Figure 7:**
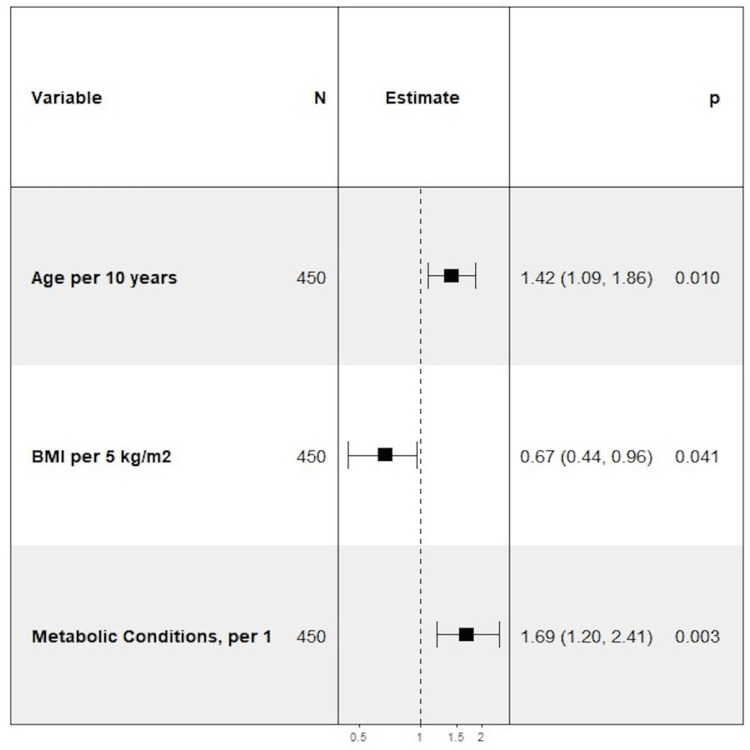
Forest Plot of Multivariable Logistic Regression Model for In-Hospital Mortality - Adjusted for Age and Body Mass Index.

On bivariable logistic regression analysis, metabolic diseases were associated with an increased likelihood of critical illness and in-hospital mortality. Amongst all variables measured, having fatty liver was the strongest predictor of in-hospital mortality (OR = 9.47, p < 0.0001). Hyperlipidemia was a significant predictor of death, p = 0.0269, whereas hypertension and type 2 diabetes approached significance, p = 0.0560 and p = 0.0605, respectively. As the number of metabolic conditions increased, the odds of critical illness (OR = 1.28, p= 0.0281) and in-hospital mortality (OR = 1.63, p = 0.0017) increased for each additional condition. Similarly, fatty liver and having four metabolic conditions were associated with an increased likelihood of the secondary composite outcome, OR = 4.13, p = 0.0108 and OR = 5.39, p = 0.0203, respectively. After adjusting for age, and BMI, the number of metabolic conditions remained significant for in-hospital mortality and approached significance for critical illness (Figures 5-7).

**Figure 8:**
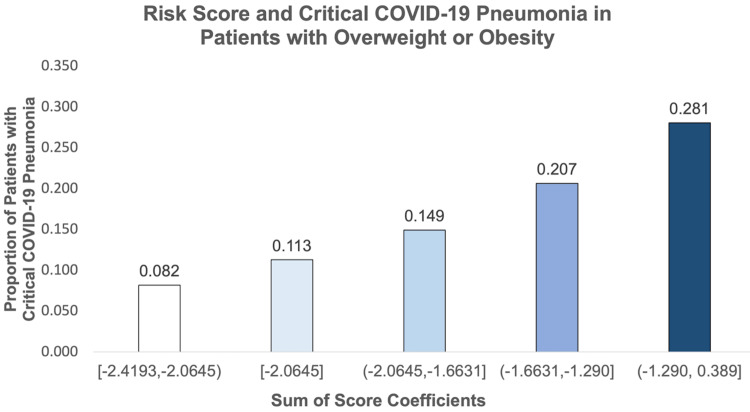
Accuracy of the Risk Score to Predict Critical COVID-19 Pneumonia in Hospitalized Patients with Overweight or Obesity COVID-19, coronavirus disease of 2019

After inputting the variables into a multivariable model, and subsequently removing those with the highest p-values, a seven-variable model was constructed for critical illness (Table 5). In this model, the two strongest predictors were age group 75 or older (OR 2.13, p = 0.019), and fatty liver (OR 4.20, p = 0.014). The sum of the score coefficients was subdivided into five score groups as shown in Figure 8. The predicted risk of critical illness in the lowest score group was 8.2% to 11.3%, and the observed risk was 8.2% (95% CI: 2.7 - 18.1%) (Table 6). In the highest risk group, the predicted risk was 23.1% to 54.4%, and the observed risk was 28.1% (95% CI: 17.0 - 41.5%). The AUROCC of the model was 0.65 (95% CI: 0.58 to 0.72).

**Table 5:** Observed Proportion of Severe COVID-19 Pneumonia According to Quintile of the Sum of the Score Coefficients COVID-19, coronavirus disease of 2019; 95% CI, 95% confidence interval

Quintiles	Proportion Severe COVID-19 Pneumonia (95% CI)
[-0.703,0.641]	0.587 (0.479, 0.698)
(0.641,1.115]	0.739 (0.651, 0.816)
(1.115,1.440]	0.800 (0.677, 0.892)
(1.440,1.790]	0.829 (0.746, 0.894)
(1.790,3.749]	0.897 (0.799, 0.959)

**Table 6:** Multivariable Logistic Regression to Derive a Risk Score to Predict Critical COVID-19 Pneumonia in Hospitalized Patients with Overweight or Obesity Calculated Predicted Risk =  2.718 ^ (sum of score coefficient) / (1 + 2.718 ^ (sum of score coefficients)) AIC: 391.44, AUROCC: 0.650 (95% CI: 0.58-0.72) OR, odds ratio; CI, confidence interval; BMI, body mass index; AIC: Akaike information criterion; AUROCC: area under the receiver operating characteristic curve

	OR (95% CI)	P Value	Score Coefficient
(Intercept)	0.13 (0.08-0.20)	< 0.001	- 2.0462
Age Group 65-74	1.12 (0.54-2.20)	0.760	+ 0.1084
Age Group 75	2.13 (1.13-4.00)	0.019	+ 0.7562
BMI 35 kg/m^2^	0.69 (0.34-1.31)	0.272	- 0.3731
Race, Asian	1.43 (0.45-3.82)	0.510	+ 0.3548
Vaccinated	1.50 (0.64-3.29)	0.330	+ 0.4050
Type 2 Diabetes	1.63 (0.91-2.87)	0.096	+ 0.4877
Fatty Liver	4.20 (1.27-13.21)	0.014	+ 1.4341

## Discussion

In our single-center cohort study of 450 patients with overweight or obesity, we found that being MU was not associated with severe COVID-19 pneumonia. However, we found that being MU was associated with a four-fold increased frequency of in-hospital mortality, despite higher rates of vaccination against SARS-CoV-2. Our risk score showed that patients with overweight and obesity have an elevated baseline risk of severe COVID-19 pneumonia of 58.7%. As the risk score increased, so did the observed rate of severe disease in our cohort. In contrast, the baseline risk for critical illness was lower at 8.2%. Finally, we found a dose-dependent association between the number of metabolic conditions and severe disease, critical illness, and in-hospital mortality.

The association between metabolic status and disease severity from COVID-19 pneumonia has not been fully studied. Limitations of prior studies have included the use of national inpatient databases, homogenous populations, and variable definitions for obesity and being MU. In a recent study from South Korea, investigators concluded that metabolic health status plays a greater role in disease severity than obesity [14]. The study utilized a national inpatient database of 4,069 patients hospitalized for COVID-19 pneumonia. Their definitions of obesity and being MU were a BMI >/= 25.0 kg/m^2^ and three or more metabolic conditions, respectively. Additionally, their definition for severe COVID-19 pneumonia did not follow the NIH guidelines. They defined severe COVID-19 as a composite of ICU admission, invasive mechanical ventilation, and ECMO, which precluded a distinction between severe and critical COVID-19 pneumonia. A similar study of 3,019 patients, published in Wuhan, China, had a higher threshold for the definition of obesity (> 28.0 kg/m^2^), and MU was defined as having at least one metabolic condition [17]. Additionally, the study followed the NIH guidelines for severe and critical COVID-19 pneumonia.

Like these prior studies, our study found that being MU was associated with an increased risk of mortality. In contrast, these studies did not evaluate the impact of MU on severe COVID-19 pneumonia. Similarly, a dose-dependent association between the number of metabolic conditions and critical illness or mortality was not explored. Fatty liver - another metabolic condition of increasing prevalence - was also not evaluated. Compared to these studies, our study had a more heterogenous population, included patients with overweight or obesity, considered MU as having at least one metabolic condition, and considerations were made to vaccination status, treatments administered, and presence of pulmonary disease. Finally, we were able to derive risk scores for severe disease and critical illness, specifically for patients with overweight and obesity.

The main finding of our study was the dose-dependent association between the number of metabolic conditions and the increased likelihood of severe COVID-19 pneumonia, critical illness, and in-hospital mortality. These associations remained for critical illness and in-hospital mortality after adjusting for age and BMI, suggesting metabolic conditions are independent of obesity. An explanation for this finding may be the concept of metabolically healthy versus metabolically unhealthy obesity [18]. Human studies have suggested that patients with obesity who are metabolically healthy (prevalence of 10-30% amongst patients with obesity) have better adipose tissue function, less ectopic fat storage, and more insulin sensitivity than MU patients [18]. Those patients who are MU have an increased level of circulating inflammatory markers, including higher levels of C-reactive protein, progranulin, and chemerin, and lower levels of adiponectin and neuregulin-4 [19]. 

A dysregulated immune response characterized by cytokine storm has been implicated as the immunopathogenesis of severe COVID-19 pneumonia [20-23]. Given patients who are MU have a higher reservoir of immunogenically active adipocytes, the observation that metabolic conditions were associated with severe disease progression of COVID-19 may be an indirect observation of the underlying degree of baseline inflammation in this patient population. Further studies with biochemical measurements of baseline inflammation are needed to elucidate this association between metabolic health and the severity of COVID-19 pneumonia.

Our study has several limitations. First, COVID-19 pneumonia was and remains a rapidly evolving disease with fast-changing treatment algorithms. Given the rapid pace of drug and vaccine development, there likely were variable treatments offered at the early onset of the pandemic compared to later in its course. Treatments, such as baricitinib and tocilizumab, and vaccinations were not available for patients before December 2021. In our cohort, less than 10% of patients were vaccinated as vaccines were only emerging by August 2021. It is unclear if higher rates and longer times from vaccination may have decreased the risk of severe COVID-19 pneumonia in this patient population. 

Second, the retrospective nature of the study limited our ability to measure other measures of metabolic disease, including waist circumference, and visceral adipose tissue, and measures of inflammation, such as C-reactive protein, procalcitonin, and interleukin-6 levels. Similarly, the retrospective design prevented us from comprehensively surveying each patient for the presence of each metabolic disorder, especially fatty liver. Given the retrospective nature, we relied on the available laboratory and imaging diagnostics to determine the presence of metabolic conditions. As such, we were unable to determine the presence of liver disease in all patients given the lack of imaging. In addition, we were unable to obtain surrogates for central adiposity, such as the waist circumference, that measure the amount of visceral adiposity.

Third, the definition of MU is not well established by expert guidelines. To improve inclusivity, we chose the more inclusive definition of having at least one metabolic comorbidity to define metabolic unhealthiness. With a stricter definition, such as having two or three metabolic conditions as the cutoff for MU, it is likely we would have found a direct association between MU and severe COVID-19 pneumonia. Nonetheless, we showed that as the number of metabolic conditions increased, so did the likelihood of severe COVID-19 pneumonia.

Fourth, the frequency of in-hospital mortality had low counts, which prevented us from deriving a risk score using multivariable logistic regression to predict death from COVID-19 pneumonia in patients with overweight and obesity. Finally, the frequency of severe COVID-19 pneumonia was relatively high in our patient population compared to previous studies. The reason for this finding is unclear but may be related to selection bias as our tertiary care center cares for medically complex patients who may have additional comorbidities that were not accounted for. Nonetheless, our study has demonstrated new and novel findings related to disease severity based on metabolic health in patients with overweight or obesity hospitalized with COVID-19 pneumonia. 

## Conclusions

Obesity is a well-recognized risk factor of severe COVID-19 pneumonia. The concept of metabolic health is emerging as a novel idea to explain the discrepant observations in variable outcomes in patients with obesity. Although obesity has been recognized as a risk factor for cardiovascular disease, diabetes, and other metabolic conditions, not every patient with obesity develops these complications. In our study, we aimed to determine the association of metabolic health with disease severity in patients hospitalized with COVID-19 pneumonia. We showed that metabolic conditions have a dose-dependent association with severe disease, critical illness, and in-hospital mortality, irrespective of age and BMI.

Although we did not show that being metabolically unhealthy was associated with disease severity, it is likely that an alternative definition for metabolic unhealthiness is needed. Our risk scores have several clinical implications, including reminding clinicians of the elevated baseline risk of severe COVID-19 pneumonia in patients with overweight or obesity, and further emphasizing the risk factors for disease severity. Taken together, our findings suggest clinicians should be aware that metabolic status plays an additive effect in severe disease progression from COVID-19 pneumonia. We recommend further validation of these risk scores in heterogenous populations with updated treatment and vaccination guidelines.

## References

[1] Umakanthan S, Sahu P, Ranade AV (2020). Origin, transmission, diagnosis and management of coronavirus disease 2019 (COVID-19). Postgrad Med J.

[2] Banerjee M, Gupta S, Sharma P, Shekhawat J, Gauba K (2020). Obesity and COVID-19: a fatal alliance. Indian J Clin Biochem.

[3] Földi M, Farkas N, Kiss S (2020). Obesity is a risk factor for developing critical condition in COVID-19 patients: a systematic review and meta-analysis. Obes Rev.

[4] Huang Y, Lu Y, Huang YM, Wang M, Ling W, Sui Y, Zhao HL (2020). Obesity in patients with COVID-19: a systematic review and meta-analysis. Metabolism.

[5] Yang J, Hu J, Zhu C (2021). Obesity aggravates COVID-19: a systematic review and meta-analysis. J Med Virol.

[6] Zhou Y, Chi J, Lv W, Wang Y (2021). Obesity and diabetes as high-risk factors for severe coronavirus disease 2019 (Covid-19). Diabetes Metab Res Rev.

[7] Liang W, Liang H, Ou L (2020). Development and validation of a clinical risk score to predict the occurrence of critical illness in hospitalized patients with COVID-19. JAMA Intern Med.

[8] Garibaldi BT, Fiksel J, Muschelli J (2021). Patient trajectories among persons hospitalized for COVID-19: a cohort study. Ann Intern Med.

[9] Goodacre S, Thomas B, Sutton L (2021). Derivation and validation of a clinical severity score for acutely ill adults with suspected COVID-19: the PRIEST observational cohort study. PLoS One.

[10] Iacobini C, Pugliese G, Blasetti Fantauzzi C, Federici M, Menini S (2019). Metabolically healthy versus metabolically unhealthy obesity. Metabolism.

[11] Blüher M (2020). Metabolically healthy obesity. Endocr Rev.

[12] Smith GI, Mittendorfer B, Klein S (2019). Metabolically healthy obesity: facts and fantasies. J Clin Invest.

[13] Tutunchi H, Naeini F, Ebrahimi-Mameghani M, Najafipour F, Mobasseri M, Ostadrahimi A (2021). Metabolically healthy and unhealthy obesity and the progression of liver fibrosis: a cross-sectional study. Clin Res Hepatol Gastroenterol.

[14] Kim NH, Kim KJ, Choi J, Kim SG (2022). Metabolically unhealthy individuals, either with obesity or not, have a higher risk of critical coronavirus disease 2019 outcomes than metabolically healthy individuals without obesity. Metabolism.

[15] Cuschieri S (2019). The STROBE guidelines. Saudi J Anaesth.

[16] (2022). Coronavirus Disease 2019 (COVID-19) Treatment Guidelines | National Institutes of Health. https://www.covid19treatmentguidelines.nih.gov/.

[17] Zeng J, Liu X, Wang S (2021). The association between BMI and metabolically unhealthy status with COVID-19 mortality: based on 3019 inpatients from Wuhan, China. Nutr Metab Cardiovasc Dis.

[18] Goossens GH (2017). The metabolic phenotype in obesity: fat mass, body fat distribution, and adipose tissue function. Obes Facts.

[19] Klöting N, Fasshauer M, Dietrich A (2010). Insulin-sensitive obesity. Am J Physiol Endocrinol Metab.

[20] Choudhary S, Sharma K, Silakari O (2021). The interplay between inflammatory pathways and COVID-19: a critical review on pathogenesis and therapeutic options. Microb Pathog.

[21] Darif D, Hammi I, Kihel A, El Idrissi Saik I, Guessous F, Akarid K (2021). The pro-inflammatory cytokines in COVID-19 pathogenesis: what goes wrong?. Microb Pathog.

[22] Hu B, Huang S, Yin L (2021). The cytokine storm and COVID-19. J Med Virol.

[23] Kim JS, Lee JY, Yang JW (2021). Immunopathogenesis and treatment of cytokine storm in COVID-19. Theranostics.

